# Coxsackievirus B detection in cases of myocarditis, myopericarditis, pericarditis and dilated cardiomyopathy in hospitalized patients

**DOI:** 10.3892/mmr.2014.2578

**Published:** 2014-09-18

**Authors:** IMED GAALOUL, SAMIRA RIABI, RAFIK HARRATH, TIMOTHY HUNTER, KHALDOUN B. HAMDA, ASSIA B. GHZALA, SALLY HUBER, MAHJOUB AOUNI

**Affiliations:** 1 Laboratory of Transmissible Diseases LR99-ES27, Faculty of Pharmacy, Monastir 5000, Tunisia; 2 DNA Microarray Facility, 305 Health Science Research Facility, University of Vermont, Burlington, VT 05405, USA; 3 Department of Pathology, University of Vermont, Burlington, VT 05405, USA; 4 Department of Cardiology, University Hospital Fattouma Bourguiba, Monastir 5000, Tunisia; 5 Department of Cardiology, University Hospitals Farhat Hached and Sahloul, Sousse 4054, Tunisia

**Keywords:** coxsackievirus B, human heart infections, molecular diagnosis, immunohistochemical investigations, epidemiology

## Abstract

Coxsackieviruses B (CV-B) are known as the most common viral cause of human heart infections. The aim of the present study was to assess the potential role of CV-B in the etiology of infectious heart disease in hospitalized patients. The present study is based on blood, pericardial fluid and heart biopsies from 102 patients and 100 control subjects. All of the samples were examined for the detection of specific enteroviral genome using the reverse transcription polymerase chain reaction (RT-PCR) and sequence analysis. Immunohistochemical investigations for the detection of the enteroviral capsid protein, VP1, from the biopsies were performed. The samples were cultured on confluent KB monolayer cell line for possible virus isolation. The epidemiological data were also collected. CV-B was detected in 28 of the 102 patients. The sequence analysis demonstrated that 27 strains were identical to CV-B3 and only one strain was identical to CV-B1. Furthermore, VP1 in the heart biopsies was detected in enterovirus-positive cases, as revealed by RT-PCR. Pericarditis infection was more frequent than myocarditis (P<0.05) or myopericarditis (P=0.05). The epidemiological data demonstrate that CV-B heart infections occur mainly during autumn and winter, and young male adults are more susceptible than adolescents or adults (P<0.5). The present findings demonstrate a higher prevalence of viral heart infections, suggesting that CV-B may significantly contribute to heart infections.

## Introduction

Cardiovascular infections include a group of entities involving the heart wall, such as myocarditis, dilated cardiomyopathy and pericarditis. These processes are associated with high morbidity and mortality. Although early diagnosis is essential for adequate patient management and leads to improved prognosis, the clinical manifestations are often non specific (1).

Myocarditis is clinically and pathologically defined as an inflammation of the heart muscle. The term myocarditis was first used in the early 19th century to describe myocardial diseases not associated with valvular abnormalities (2), but only in the second half of the 20th century was interest in inflammatory myocardial diseases renewed (3). A number of patients with acute viral myocarditis may develop dilated cardiomyopathy as a complication (4–19). Patients who have suffered from a heart attack may develop pericarditis over the subsequent days or weeks. Pericarditis is a swelling and irritation of the pericardium, the thin sac-like membrane that surrounds the heart. It is most commonly sudden and acute. When the symptoms develop more gradually or persist, pericarditis is considered chronic (1,20,21).

Acute pericarditis and myocarditis often occur together although they are rarely of the same intensity (22,23). When both are present, they generally trigger clinical syndromes that are mainly pericarditic or myocarditic (24). The term myopericarditis indicates a primarily pericarditic syndrome with minor myocardial involvement, which describes the majority of combined pericarditis and myocarditis cases encountered in clinical practice. By contrast, the term perimyocarditis indicates a primarily myocarditic syndrome. However, these two terms are often used interchangeably without regard to the predominant type of cardiac involvement (22,25).

Myocarditis, with or without pericarditis, is becoming an increasingly common diagnosis. Numerous agents are known to cause these heart infections and viruses are considered to be the most important causative agent. Coxsackieviruses B (CV-B) have been involved in 25–40% cases of acute myocarditis and dilated cardiomyopathy in infants and young adolescents (26–28). CV-B belong to the enterovirus group of the Picornaviridae family and are the causative agents of a broad spectrum of clinically relevant diseases, including acute and chronic myocarditis, meningitis and possibly autoimmune diabetes (29). The 7.4 kb positive stranded RNA genome of CV-B consists of a 5′-untranslated region (UTR) followed by a single polyprotein coding region and a 3′-UTR, flanked by a poly A-tail. The first part of the polyprotein (P1) encodes the four capsid proteins while the second and third part (P2 and P3, respectively) encode non-structural proteins involved in genome processing and RNA synthesis. The four capsid proteins, VP1-VP4, are grouped into a pseudo-icosahedral capsid. The VP1–VP3 constitute the outer surface of the viral particle, whilst VP4 is embedded within the inner surface of the capsid (30). Outbreaks of myocarditis most commonly occur in young children, however sporadic cases are observed in older children and adults (31–34).

Studies on enterovirus infections in heart muscle disease have been promoted, by methods using the reverse transcriptase-polymerase chain reaction. As a result of this technique, the enteroviral genomic RNA was detected in samples of patients with infectious heart diseases (9,14). However, when a low copy number of viruses is present in the samples, the RT-PCR may fail to produce enough amplified products (amplicons) to be either detected by the ethidium bromide staining or used for further molecular manipulation (35).

In the present study, the involvement of CV-B as an etiological agent in infectious heart diseases was investigated by the detection of the genomic RNA in blood and pericardial fluid samples from patients suffering from myocardial and/or pericardial diseases. Immunohistochemical investigations for the detection of the enteroviral capsid protein, VP1, from heart biopsies were also performed. Cell cultures for possible virus isolation followed by indirect immunofluorescence assay (IFA) for the detection of VP1 capsid protein were also conducted. In addition, the epidemiological data for CV-B heart infections were described. This information was collected from Tunisian hospitalized patients over five years (2006–2011) upon admission to the Cardiology Departments at Fattouma Bourguiba (Monastir, Tunisia) and Sahloul (Sousse, Tunisia) hospitals.

## Materials and methods

### Patients

The present study reports 102 patients (89 males and 13 females) clinically selected for presentation with inflammatory heart diseases, and aged between 17 and 46 years (mean age 30.8 years). Myocardium and/or pericardium inflammation was presumed to be due to a typical history of viral infection at the time of cardiac disease onset. A number of clinical signs of viral impregnation, including rhinorrhea, coughing, myalgia, muscle soreness, high fever as well as cardiac symptoms, such as chest pain, breathlessness, cardiac arrhythmia, easy fatigability, electrocardiographic changes and reduced exercise, were observed. In terms of susceptibility and history, the patients are most commonly adolescents and young adults. Further biological tests were performed. In fact, a complete blood count marked by a hyperleukosytosis with a lymphocyte predominance, marginally raised C-reactive protein and procalcitonin, supported the suspicion of a viral heart infection. The samples were obtained from patients within 48 h following admission to the Cardiology Departments at Fattouma Bourguiba and Sahloul hospitals. The sampling was performed at three university hospitals located on the coast of Tunisia: Fattouma Bourguiba (Monastir), Farhat Hached (Sousse) and Sahloul (Sousse) between November 2006 and November 2011. A total of 100 adult subjects presenting with other cardiac pathologies than infectious ones were used as the controls. The study was approved by the ethics committees of the University Hospitals Fattouma Bourguiba (Monastir, Tunisia), Farhat Hached and Sahloul (Sousse, Tunisia). Each Ethic Committee is designed by the hospital where the sampling was performed. Written informed consent forms were signed by patients and the controls participating in this study.

### Reverse transcriptase-polymerase chain reaction (RT-PCR)

The viral RNA was extracted from blood, pericardial fluid samples using the TRIzol^®^ Plus RNA Purification kit (Invitrogen Life Technologies) according to the manufacturer’s instructions. DNase treatment during RNA purification was adopted using the PureLink™ DNase (Invitrogen Life Technologies) in order to obtain DNA-free total RNA. One-step RT-PCR for the detection of enterovirus RNA was performed with primers directed to the conserved sequences in the 5′-UTR of the enterovirus genome. A fragment of 155 bp of the extracted RNA was amplified by one-step RT-PCR (Invitrogen SuperScript™ One-Step RT-PCR with Platinum^®^ Taq) using 006 and 007 primers (36). The RT-PCR was performed on a mixture containing 25 μl 2X reaction mix (a buffer containing 0.4 mm of each dNTP; 2.4 mm MgSO_4_); 0.2 μM each of sense and anti-sense primers, 1 μl of enzyme mix (RT/Platinum^®^ Taq; Invitrogen Life Technologies) and RNase free water to 50 μl. The reaction was conducted with an initial reverse transcription step at 42°C for 30 min, followed by PCR activation at 94°C for 5 min, 30 amplification cycles (94°C, 30 sec; 42°C, 1 min; 72°C, 2 min) and a final 10-min extension at 72°C in an Eppendorf Mastercycler Thermal Cycler. The PCR products were run on a 2% agarose gel stained with ethidium bromide and visualized under UV light.

### Sequencing and analysis of PCR enterovirus amplification products

First, the PCR amplicons were purified using the ExoSAP-IT-PCR Clean-Up Reagent (USB^®^ Products from Affymetrix, Inc) which constitutes a one-step enzymatic cleanup of PCR products. Next, they were sequenced in forward and reverse directions with the respective PCR primers. The chromatogram sequencing files were inspected with Finch TV (version 1.4.0). The obtained enterovirus sequences were compared with the corresponding ones available in the GenBank using Basic Local Alignment Search Tool (BLAST) in order to identify the enterovirus type (37,38).

### Histopathology: hematoxylin-eosin staining

Heart biopsies obtained from the patients and controls were fixed in formalin (neutral-buffered formaldehyde, 30% diluted to 1/10) for 24 h and embedded in paraffin. The sections (5 μm) were cut from the paraffin-embedded tissues with a microtome. All of the sections were stained with hematoxylin-eosin staining (Invitrogen Life Technologies) and the slides were investigated for the presence of infectious markers (39–42).

### Immunohistochemical analysis

In all the heart biopsies, immunohistochemical investigations were performed on the enteroviral capsid protein VP1 Mab 5-D8/1 (DAKO, Carpenteria, VT, USA). Tris-buffered NaCl solution with Tween-20, the target retrieval solution, serum-free protein block, antibody diluent, mayer’s hematoxylin, EnVision^+^ system-HRP (AEC) and Glycergel^®^ Mounting Medium (aqueous) were all purchased from DAKO. The immunohistochemical procedures included antigen exposure, blocking, incubation with primary antibody, incubation with the secondary antibody in the En-Vision detection system and appropriate wash between steps using Tris-buffered saline with Tween-20. All the incubations were performed at room temperature (5,43–45). Briefly, paraffin-embedded tissue sections (5 μm) were dewaxed with xylene and rehydrated with graded ethanol. Antigen exposure was achieved by heat in a water bath (95–99°C) mediated by target retrieval. The endogenous peroxidase activity was blocked with a peroxidase-blocking reagent for 15 min. The tissue sections were blocked with the protein block for 10 min and then incubated with primary antibody (appropriately diluted to 1:100–1:500 in antibody diluent) for 30 min and washed. The secondary antibody in the En-Vision detection system was the goat anti-mouse Ig conjugated with dextran polymer, on which numerous peroxidase molecules were labeled. The sections were incubated with this reagent for 30 min, washed and then reacted with substrate chromogen for 5–10 min. The slides were immersed in aqueous hematoxylin for counterstaining. The color reaction was stopped by a wash in distilled water. Finally, the mounted sections were examined and confirmed under a Nikon Eclipse 50i microscope (Nikon, Tokyo, Japan).

### Virus isolation by in vitro cell culture

Virus isolation was performed on KB cells (human squamous carcinoma cell line). The cells were grown in 24-well microplates in RPMI-1640 medium (Invitrogen Life Technologies), washed twice with phosphate-buffered saline (PBS) and then inoculated with 50 μl of the patients’ samples (blood and pericardial fluid). The samples were allowed to adsorb for 1 h at 37°C with gentle swirling every 15 min. Eagle’s minimal essential medium with 5% fetal calf serum, 100 U/ml penicillin, 200 μg/ml streptomycin and 1 mg/ml l-glutamine (Gibco-BRL, Invitrogen Life Technologies) were then added to the infected cells. The cells were monitored daily for characteristic cytopathic effect (CPE) (6).

### Indirect immunofluorescence assay (IFA)

To detect viral internalization, the cells were fixed in cold acetone (15 min) and subjected to an indirect immunofluorescence assay intended to detect the VP1 capsid protein. Briefly, the slides were incubated with enterovirus group specific monoclonal antibody (mAb) 5-D8/1 (diluted at 1/40 in PBS; Dako, Trappes, France) for 30 min at room temperature. Following three washes in PBS, fluorescein isothiocyanate (FITC)-conjugated goat anti-mouse antibody (Jackson ImmnuoResearch Laboratories, Inc., West Grove, PA, USA) was applied at a dilution of 1:25 in blue Evans for a further 30 min at room temperature. Subsequently, the slides were washed again with PBS, dried, mounted with glycerol medium and examined using a fluorescence microscope (Olympus, Rungis, France) (5).

### Statistical analysis

To determine the risk groups, a statistical analysis (chi-squared test) with a 95% significance level was performed using Epi Info version 6.04 (CDC, Atlanta, GA, USA).

## Results

### Enterovirus RNA detection by RT-PCR and epidemiological characteristics

**Figure 1:**
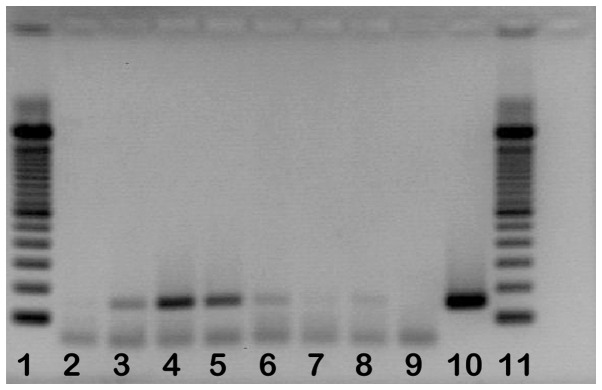
Agarose gel analysis of coxsackievirus B RNA amplified by one step reverse transcriptase-polymerase chain reaction (Lanes 1 and 11, molecular size marker 100 bp DNA ladder; lane 2, negative control; lanes 3 to 6 and 8, samples from coxsackievirus B positive cases; lanes 7 and 9, samples from a coxsackievirus B negative case; lane 10, a positive control; coxsackievirus B3: 155 bp).

Of the 102 evaluated affected individuals, 51 patients were clinically diagnosed with myocarditis, 31 with pericarditis, 12 with myopericarditis and 8 with dilated cardiomyopathy. The patients were divided into groups according to age: adolescents (13–18 years old; n=23), young adults (19–29 years old; n=63) and adults (aged >29 years; n=16). The samples were tested for the presence of genomic RNA by RT-PCR using primers located in the 5′-UTR of the enterovirus genome. The RT-PCR amplified the enteroviral genome in 9 of 51 samples obtained from myocarditis patients, in 15 of 31 samples obtained from pericarditis patients, in 2 of 12 samples obtained from myopericarditis patients and in 2 of 8 samples obtained from dilated cardiomyopathy patients. Pericarditis infection was then more frequent than myocarditis (P<0.05) or myopericarditis (P=0.05). Of the 28 positive RT-PCR samples, 17 were simultaneously positive in blood and pericardial fluid samples. Fig. 1 shows an example of detection by agarose gel electrophoresis after RT-PCR. However, of the 100 control samples, none were RT-PCR positive.

**Figure 2:**
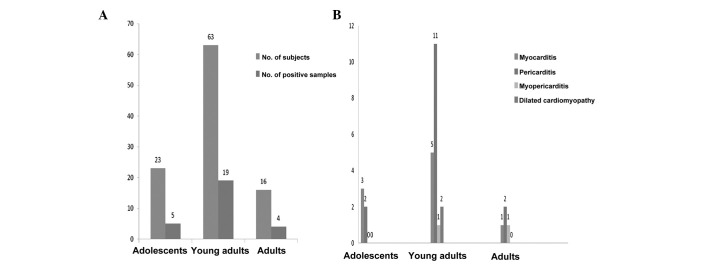
(A) Proportions and (B) clinical distribution of hospitalized patients testing positive for coxsackieviruses B heart infections according to the age.

**Figure 3:**
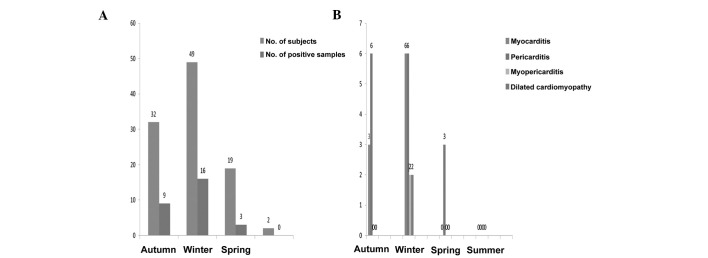
(A) Proportions and (B) clinical distribution of hospitalized patients testing positive for coxsackieviruses B heart infections according to the seasonality.

**Figure 4:**
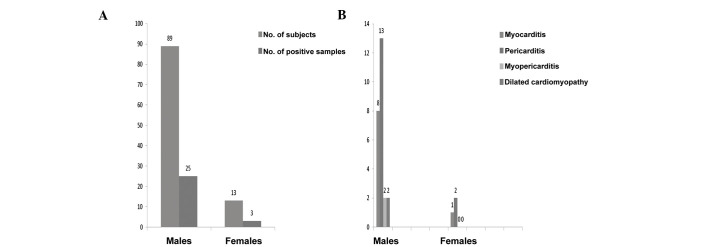
(A) Proportions and (B) clinical distribution of hospitalized patients testing positive for coxsackieviruses B heart infections according to the sex.

When analyzed according to age group, the differences in outcome were notable between groups (Fig. 2). Five out of 23 adolescents, 19 out of 63 young adults and 4 out of 16 adults tested positive for enteroviral RNA by RT-PCR. Infection was higher in young adults than in adults (P<0.5) or adolescents (P<0.5). When analyzed by seasonal variation, the differences in outcome were also significant. Fig. 3 demonstrates the following distribution: enteroviral RT-PCR was positive in 9 of 32 in autumn, 16 of 49 in winter and 3 of 19 in spring. The analysis of the enteroviral RT-PCR positivity according to gender shows the following distribution: 25 of 89 males and 3 of 13 females were positive. Thus, males seem more susceptible (P<0.5; Fig. 4).

### Sequence analysis

The sequence analysis of enterovirus amplimers showed that 27 strains were identical to the CV-B3 sequence in 9 myocarditis cases, 15 pericarditis cases, 1 myopericarditis case and 2 dilated cardiomyopathy cases. Only one strain was identical to the CV-B1 sequence in 1 myopericarditis case.

### Histopathological investigation

**Figure 5:**
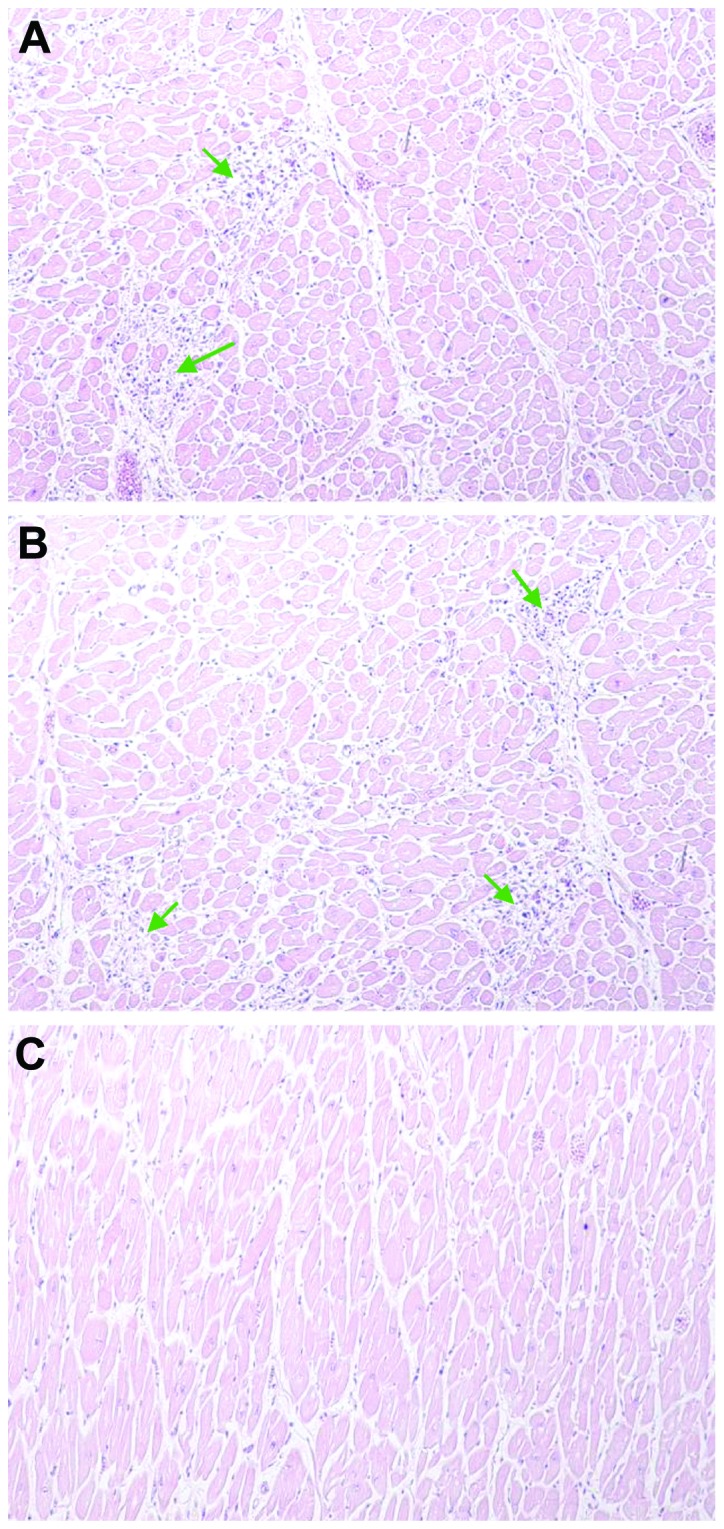
Histological specimen (hematoxylin-eosin staining). (A and B) Areas of diffuse inflammation; (C) control samples demonstrating no significant pathological findings.

Following investigation of the paraffin tissue blocks from patients, diffuse inflammation was marked (Fig. 5A and B). The slides from the paraffin tissue blocks from the controls demonstrated no significant pathological findings (Fig. 5C).

### Immunohistochemical detection of enteroviral capsid protein VP1

**Figure 6:**
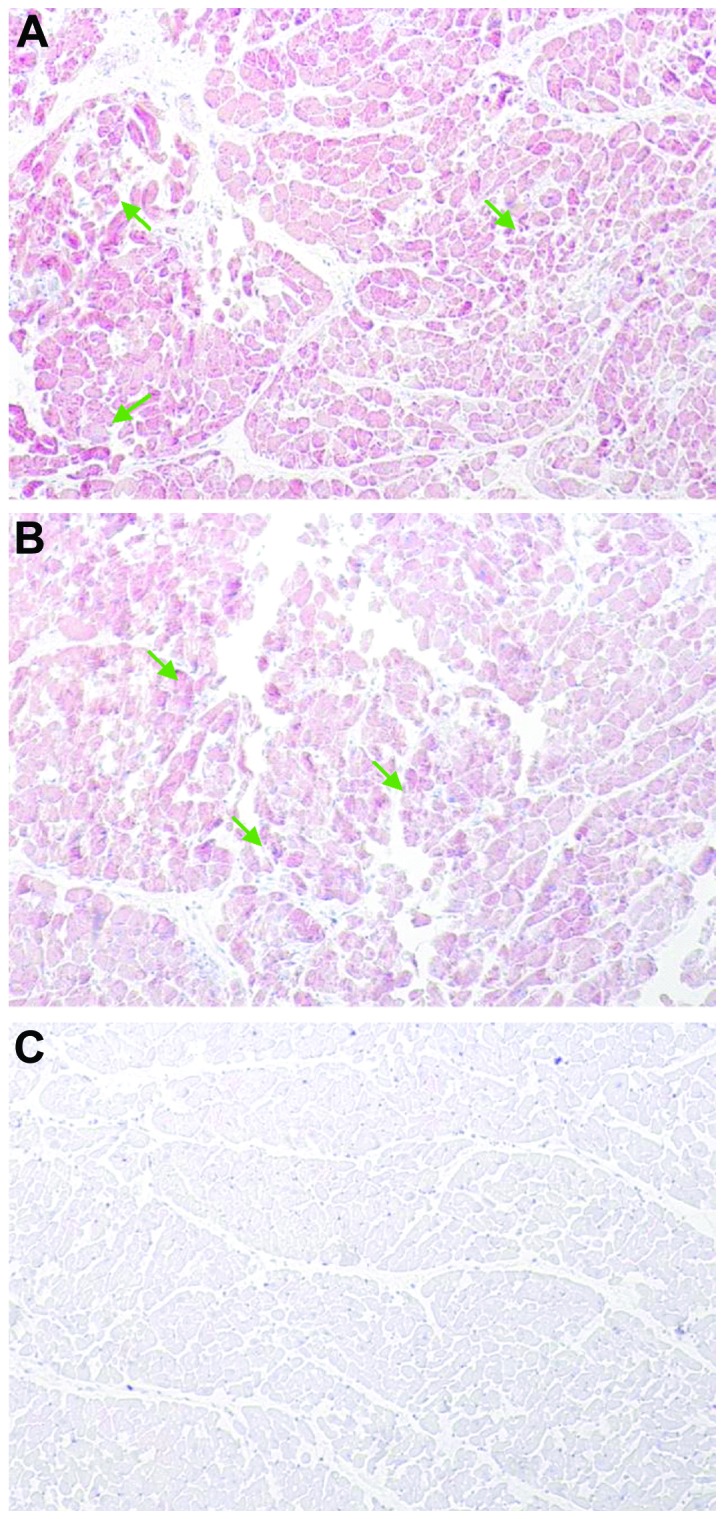
Immunohistochemical detection of enteroviral capsid protein VP1 in biopsies samples. (A and B) Enteroviral capsid protein VP1 detected suggesting a confluent invasion of enterovirus. (C) Control samples demonstrating no significant pathological findings.

To determine whether the enterovirus cardiac infection was associated with a viral protein synthesis activity, all of the heart biopsies obtained from hospitalized patients and controls were examined by immunohistochemical assays specific for the enteroviral capsid protein VP1. The VP1 capsid protein in the heart biopsies was detected in enterovirus-positive cases revealed by means of RT-PCR (Fig. 6A and B). The slides from the control subjects demonstrated a negative result with no significant pathological findings (Fig. 6C).

### Culture and indirect immunofluorescence assay detection of VP1 enterovirus

**Figure 7:**
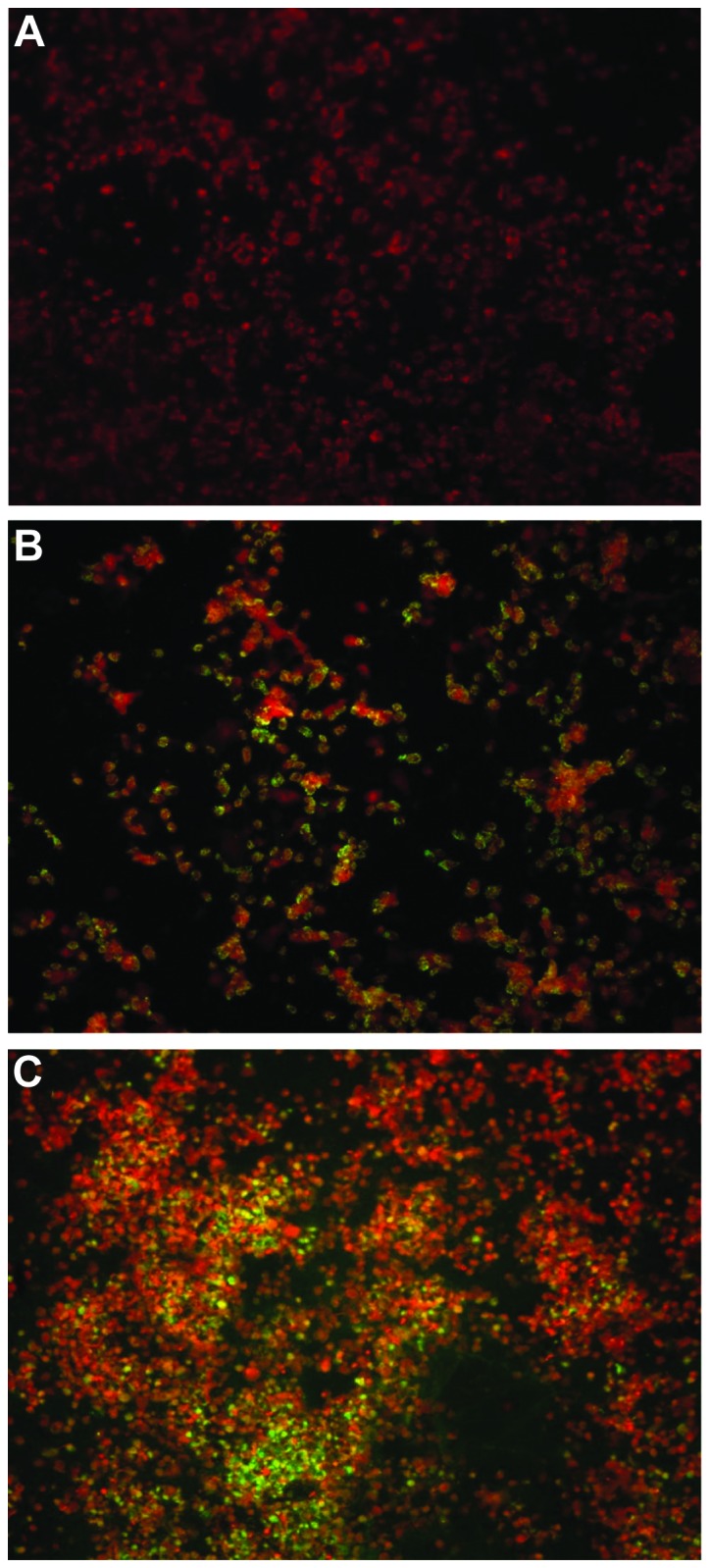
Detection of enterovirus VP1 protein by immunofluorescence staining in KB cell line. (A) Negative control (non-infected cells). (B) KB cells infected by coxsackieviruses B. VP1-positive cells (green). VP1-negative cells (red). (C) Positive control (cells infected with Nancy prototype).

Enterovirus was cultured from 1 of 43 pericardial fluid samples whereas no positive viral cultures were found in all of the blood samples (n=102). Within the control group, enteroviral cell culture was negative. The sample demonstrating a positive cytopathic effect in cell culture was also positive for VP1 antigen detection by immunostaining assay (Fig. 7).

## Discussion

The present study, conducted in the coastal region of Tunisia, aimed to investigate the presence of enterovirus markers of infection (genomic RNA or VP1 antigen) in blood, pericardial fluid samples and heart biopsies in hospitalized patients suffering from myocardial and/or pericardial heart diseases. Enteroviral genomic RNA was identified in 28 cases (27.45%) using the RT-PCR assays. Having demonstrated that enteroviral RNA is often involved in different forms of infectious heart diseases, sequencing was adopted to confirm the involvement of these viruses.

As an enterovirus group-specific monoclonal antibody (Mab) is now available, the demonstration of enteroviral antigens in heart tissue is possible (17). In the present study, this Mab was used to detect enteroviral antigens (VP1) in formalin-fixed heart biopsies, paraffin-embedded sections. Using immunohistochemistry, the enteroviral capsid protein VP1 in the heart biopsies was detected in all enterovirus-positive cases revealed by means of RT-PCR. In the present study, the sequence analysis demonstrated that CVB-3 and CVB-1 are the most implicated in heart infections. Similarly, RT-PCR as well as immunohistochemistry revealed an association between enterovirus infection, in particular the cardiotropic coxsackie B enterovirus, serotypes B1-B5 and myocarditis (46). In the diagnostic approach, the positive results were confirmed using a variety of techniques. The frequency and identification of infectious agents with infectious heart diseases, including myocarditis, have varied widely between 10 and 100% (27,47–49). These marked variations between studies are due to several factors, including the studied samples (blood, cardiac puncture, pericardial fluid samples, heart biopsies), the methods used to detect the infectious agent, result confirmation, correlation with clinical and histological findings, and the time during the illness when the samples were obtained.

In the present study, the series of 51 myocarditis, 31 pericarditis, 12 myopericarditis and 8 dilated cardiomyopathy cases suspected clinically, blood, pericardial fluid samples and heart biopsies were used according to a pre-set protocol. RT-PCR complemented with sequencing and histopathology supplemented with immunohistochemistry were the methods that could be performed to detect the infectious agents.

Enterovirus isolation in cell culture and detection of their cytopathic effect were adopted in the present study. Enterovirus was cultured in the pericardial fluid sample from one pericarditis patient. Of higher significance, 25–35% of the specimens from the patients with characteristic enterovirus infection of any serotype will be negative by cell culture due to antibody neutralization *in situ*, inadequate sample collection, handling or processing of the samples and cell line intrinsic insensitivity (50). The failure to detect enteroviral infection markers is not infrequent and has generated several hypotheses, including antigen mimicry and efficient clearing of the organism by the time of testing and autoimmune reactions (27,47,51). It may be hypothesized that a number of the microorganism-negative cases, particularly in adults, are non-infectious in nature and are due to drugs and toxins. Furthermore, limitations of the assays may also be responsible for the inability to make an organism-specific diagnosis in every myocarditis case (47). However, a higher prevalence of viral cardiopathy may be revealed by the application of a comprehensive combination of molecular pathological analysis for the detection of enterovirus RNA from blood and pericardial fluid samples, and immunohistochemical techniques for the detection of the enteroviral capsid protein VP1 from formalin-fixed, paraffin-embedded heart tissue.

The present epidemiological study has provided some new results of marked interest. Age, gender and seasonal variation appear to be important factors affecting the CV-B prevalence in human infectious heart diseases. Although enterovirus infections in Tunisia generally reach a peak in autumn (52), this seasonal variation was also found to progress until winter, as demonstrated in the present study. According to age, CV-B specifically affects young male adults, possibly because they have a more intense and active lifestyle. Finally, males appear to be more susceptible to these infections, which may raise the hypothesis of different hormone secretions. To the best of our knowledge, the present study is the first report of the epidemiology of CV-B heart infections in Tunisia. These viruses are associated with various diseases and epidemiological data may help to clarify their impact on human health.

In conclusion, the present study suggests that the pathogenesis of myocardial and/or pericardial diseases is highly associated with CV-B infection. The combined investigations using molecular pathological techniques and immunohistochemical methods has improved our ability to diagnose viral heart infections in a rapid and specific way. Such a prompt diagnosis has implications in the treatment of patients. These findings may be of major interest for the development of further therapies or preventive strategies in the prevention of the development of cardiovascular infections and inflammations. Further studies are required to determine other virus serotypes involved in these heart infections. Prospective murine experimental myocarditis may be desirable and may have a marked impact on the quality of future investigations to improve the understanding and elucidate the physio and immuno-pathogenesis of such infectious heart diseases.

## References

[1] Ben-Haim S, Gacinovic S, Israel O (2009). Cardiovascular infection and inflammation. Semin Nucl Med.

[2] Mattingly TW (1965). Changing concepts of myocardial diseases. JAMA.

[3] Kaski JP, Burch M (2007). Viral myocarditis in childhood. Paediatr Child Health.

[4] Dörner A, Pauschinger M, Schwimmbeck PL, Kühl U, Schultheiss HP (2000). The shift in the myocardial adenine nucleotide translocator isoform expression pattern is associated with an enteroviral infection in the absence of an active T-cell dependent immune response in human inflammatory heart disease. J Am Coll Cardiol.

[5] Zhang H, Li Y, Peng T (2000). Localization of enteroviral antigen in myocardium and other tissues from patients with heart muscle disease by an improved immunohistochemical technique. J Histochem Cytochem.

[6] Peng T, Li Y, Yang Y (2000). Characterization of enterovirus isolates from patients with heart muscle disease in a selenium-deficient area of China. J Clin Microbiol.

[7] Bowles NE, Olsen EGJ, Richardson PJ, Archarda LC (1986). Detection of Coxsackie-B-virus-specific RNA sequences in myocardial biopsy samples from patients with myocarditis and dilated cardiomyopathy. Lancet.

[8] Pauschinger M, Phan MD, Doerner A, Kuehl U (1999). Enteroviral RNA replication in the myocardium of patients with left ventricular dysfunction and clinically suspected myocarditis. Circulation.

[9] Andreoletti L, Hober D, Decoene C (1996). Detection of enterovirus RNA by polymerase chain reaction in endomyocardial tissue of patients with chronic diseases. J Med Virol.

[10] Maekawa Y, Ouzounian M, Opavsky MA, Liu PP (2007). Connecting the missing link between dilated cardiomyopathy and viral myocarditis: virus, cytoskeleton, and innate immunity. Circulation.

[11] Petitjean J, Freymuth F, Kopecka H (1994). Entérovirus et cardiomyopathies. Méd Mal Infect.

[12] Kaski JP, Burch M (2007). Viral myocarditis in Childhood. Pediatr Child Health.

[13] Dan M, Chantler JK (2005). A genetically engineered attenuated coxsackievirus B3 strain protects mice against lethal infection. J Virol.

[14] Mariani M, Petronio AS, Manes MT (1996). Detection of enteroviral infection in myocardial tissues by polymerase chain reaction (PCR). Clin Microbiol Infect.

[15] Kandolf R, Klingel K, Zell R (1993). Molecular mechanisms in the pathogenesis of enteroviral heart disease: acute and persistent infections. Clin Immunol Immunopathol.

[16] Muir P (1992). The association of enteroviruses with chronic heart disease. Med Virol.

[17] Li Y, Bourlet T, Andreoletti L (2000). Enteroviral capsid protein VP1 is present in myocardial tissues from some patients with myocarditis or dilated cardiomyopathy. Circulation.

[18] Pauschinger M, Chandrasekharan K, Noutsias M (2004). Viral heart disease: molecular diagnosis, clinical prognosis, and treatment strategies. Med Microbiol Immunol.

[19] D’Ambrosio A, Patti G, Manzoli A (2001). The fate of acute myocarditis between spontaneous improvement and evolution to dilated cardiomyopathy: a review. Heart.

[20] Goyle KK, Walling AD (2002). Diagnosing pericarditis. Am Fam Physician.

[21] Ferreira AG, Ferreira SM, Gomes ML, Linhares AC (1995). Enteroviruses as a possible cause of myocarditis, pericarditis and dilated cardiomyopathy in Belém, Brazil. Braz J Med Biol Res.

[22] Imazio M, Trinchero R (2008). Myopericarditis: Etiology, management, and prognosis. Int J Cardiol.

[23] Smith WG (1970). Coxsackie B myopericarditis in adults. Am Heart J.

[24] Imazio M, Trinchero R (2010). The spectrum of inflammatory myopericardial diseases. Int J Cardiol.

[25] Imazio M, Cecchi E, Demichelis B (2008). Myopericarditis versus viral or idiopathic acute pericarditis. Heart.

[26] Leonard EG (2004). Viral myocarditis. Pediatr Infect Dis J.

[27] Feldman AM, McNamara D (2000). Myocarditis. N Engl J Med.

[28] Kandolf R, Ameis D, Kirschner P, Canu A (1987). In situ detection of enteroviral genomes in myocardial cells by nucleic acid hybridization: an approach to the diagnosis of viral heart disease. Proc Natl Acad Sci USA.

[29] Klingel K, Rieger P, Mall G (1998). Visualization of enteroviral replication in myocardial tissue by ultrastructural *in situ* hybridization: identification of target cells and cytopathic effects. Lab Invest.

[30] Rueckert RR, Fields BM, Knipe DM, Howley PM (1996). Picornaviridae: The viruses and their replication. Fields Virology.

[31] Dettmeyer R, Baasner A, Schlamann M, Haag C, Madea B (2002). Coxsackie B3 myocarditis in 4 cases of suspected sudden infant death syndrome: diagnosis by immunohistochemical and molecular-pathologic investigations. Pathol Res Pract.

[32] Esfandiarei M, Suarez A, Amaral A (2006). Novel role for integrin-linked kinase in modulation of coxsackievirus B3 replication and virus-induced cardiomyocyte injury. Circ Res.

[33] Ahn J, Joo CH, Seo I (2005). All CVB, serotypes and clinical isolates induce irreversible cytopathic effects in primary cardiomyocytes. J Med Virol.

[34] Treacy A, Carr MJ, Dunford L (2010). First report of sudden death due to myocarditis caused by adenovirus serotype. J Clin Microbiol.

[35] Zhang H, Soteriou B, Knowlson S, Theodoridou A, Archard LC (1997). Characterisation of genomic RNA of Coxsackievirus B3 in murine myocarditis: reliability of direct sequencing of reverse transcription-nested polymerase chain reaction products. J Virol Methods.

[36] Zoll GJ, Melchers WJ, Kopecka H (1992). General primer-mediated polymerase chain reaction for detection of enteroviruses: application for diagnostic routine and persistent infections. J Clin Microbiol.

[37] Altschul SF, Gish W, Miller W, Myers EW, Lipman DJ (1990). Basic local alignment search tool. J Mol Biol.

[38] Oberste MS, Maher K, Flemister MR (2000). Comparison of classic and molecular approaches for the identification of untypeable enteroviruses. J Clin Microbiol.

[39] Cioc AM, Nuovo GJ (2001). Histologic and in situ viral findings in the myocardium in cases of sudden, unexpected death. Mod Pathol.

[40] Dettmeyer R, Kandolf R, Schmidt P, Schlamann M, Madea B (2001). Lympho monocytic enteroviral myocarditis traditional, immunohistological and molecularpathological methods for diagnosis in a case of suspected sudden infant death syndrome (SIDS). Forensic Sci Int.

[41] Guarner J, Bhatnagar J, Shieh WJ (2007). Histopathologic, immunohistochemical, and polymerase chain reaction assays in the study of cases with fatal sporadic myocarditis. Hum Pathol.

[42] Chimenti C, Frustaci A (2008). Histopathology of myocarditis. Diagn Histopathol.

[43] Shi SR, Key ME, Kalra KL (1991). Antigen retrieval in formalinfixed paraffin-embedded tissues: an enhancement method for immunohistochemical staining based on microwave oven heating of tissue sections. J Histochem Cytochem.

[44] Shi SR, Cote RJ, Taylor CR (1997). Antigen retrieval immunohistochemistry: past, present and future. J Histochem Cytochem.

[45] Sabattini E, Bisgard K, Ascani SS, Poggi (1998). The EnVision^++^ system: a new immunohistochemical method for diagnostics and research: critical comparison with the APAAP, ChemMate, CSA, LABC and SABC techniques. J Clin Pathol.

[46] Jin O, Sole MJ, Butany JW (1990). Detection of enterovirus RNA in myocardial biopsies from patients with myocarditis and cardiomyopathy using gene amplification by polymerase chain reaction. Circulation.

[47] Guarner J, Bhatnagar J, Shieh WJ (2007). Histopathologic, immunohistochemical, and polymerase chain reaction assays in the study of cases with fatal sporadic myocarditis. Hum Pathol.

[48] Bowles NE, Ni J, Kearney DL (2003). Detection of viruses in myocardial tissues by polymerase chain reaction. Evidence of adenovirus as a common cause of myocarditis in children and adults. J Am Coll Cardiol.

[49] Cambridge M, MacArthur C, Waterson A, Goodwin J, Oakley C (1979). Antibodies to Coxsackie B viruses in congestive cardiomyopathy. Br Heart J.

[50] Abzug MJ, Rotbart HA (1999). Enterovirus infections of neonates and infants. Semin Pediatr Infect Dis.

[51] Peters NS, Poole-Wilson PA (1991). Myocarditis - continuing clinical and pathologic confusion. Am Heart J.

[52] Bahri O, Rezig D, Ben Nejma-Oueslati B (2005). Enteroviruses in Tunisia: virological surveillance over 12 years (1992–2003). J Med Microbiol.

